# The impact of large-scale sports events on national identity: a structural equation model based on the residents' perspective

**DOI:** 10.3389/fspor.2024.1479425

**Published:** 2025-01-07

**Authors:** Jing Wang, Zhiyi He, Yijuan Lu

**Affiliations:** School of Physical Education, Hangzhou Normal University, Hangzhou, Zhejiang, China

**Keywords:** large-scale sports events, Asian Games, subjective well-being, city image, national identity

## Abstract

**Introduction:**

Large-scale sports events have a profound impact on the enhancement of residents' national identity. The study takes the Hangzhou Asian Games as an opportunity to investigate the influence of such events on national identity and the underlying factors.

**Methods:**

The study takes the impact of residents' involvement in the Asian Games on their national identity as the research object, constructs a theoretical model with subjective well-being and city image as the mediator variables, randomly selects 1,096 residents by questionnaire survey, and analyzes their interrelationships as well as deeper influencing factors by using structural equation modeling and bootstrap.

**Results:**

The results show that the involvement of Hangzhou residents in the Asian Games has a significant positive impact on national identity, subjective well-being and city image (Estimate = 0.237/0.287/0.3). Furthermore, subjective well-being and city image have a significant positive impact on national identity (Estimate = 0.321/0.141), and city image has a significant positive effect on subjective well-being (Estimate = 0.264).

**Discussion:**

It can be concluded that the degree of involvement in major sports events has a direct positive effect on national identity, and subjective well-being and city image play a partially mediating role in this effect. The results of the study provide a resident's perspective for assessing the impact of large-scale sports events, and provide elements that can be used as a reference for governments, city planners and event organizers to assess large-scale sports events.

## 1 Introduction

As globalization continues to accelerate and deepen, global production networks have weakened the power of national economic discourses. Increased intercultural exchanges and interactions have led to dilemmas of individual identity and triggered a crisis of national identity. Furthermore, local conflicts continue to fester, and the world order is turbulent and unstated, making the promotion of national identity a necessity of the international situation.

It has become evident that large-scale sports events can have a profound impact on countries, cities and residents (1–3). As one of the primary political socialization functions of sport, large-scale sporting events have a significant impact on disseminating the national image, safeguarding territorial integrity and consolidating the national identity and sense of belonging (4). The Olympics is an important channel for transmitting values across social boundaries such as ethnicity, religion, politics, class, etc. Large-scale sports events can also have impacts on many aspects of society, economy, and residents' lives, and these impacts may be one of the mediating factors in their influence on residents' national identity. Unanue's survey found that large-scale sports events have a significant impact on spectators' subjective well-being and confirmed the correlation between subjective well-being and national identity (5); Huang et al.'s survey found that “city popularity and image” is the most weighted economic impact of sports events (6); Huang et al. found that iconic sports events have an effect on the city's “infrastructure”, “visual image” and “cultural image” (7), and that a good city image can also increase the pride of residents and stimulate cohesion. This suggests that happiness and city image play a role in the influence of sports events on national identity, and that research on them can provide insights into the specific extent and path of the influence of large-scale sports events on residents' national identity.

These previous studies provide an important research foundation for exploring the impact of large-scale sports events on residents' national identity. However, the existing studies are more experimental than empirical, focusing mostly on the direct impact of sports events on national identity and neglecting the complexity and indirectness of the impact among different factors. There are fewer studies on the construction of multi-factor influence models, neglecting the deep multi-dimensional study of influence paths.

The 19th Asian Games Hangzhou (Hereinafter referred to as “Hangzhou Asian Games”) was held on 23 September 2023 in Hangzhou, Zhejiang Province, China. This research takes the hosting status as an opportunity to examine the overall social function of large-scale sports events. It incorporates the host city and the participation of residents into the research logic of influencing national identity. It explores the mechanisms and pathways through which large-scale sports events can promote national identity, providing direction and suggestions for enhancing residents' subjective well-being and promoting national identity. Furthermore, it serves as a basis for evaluating the social benefits of large-scale sports events and improving their social impact.

### 1.1 Involvement

The concept of involvement was first introduced by scholars Sherif & Cantril in the field of social psychology to evaluate a person's attitudes and behaviors in participating in something (8). Involvement can be defined as the level of personal relevance and psychological connection that reflects a person's level of commitment, perceived personal relevance and psychological connection to a goal, action or experience (9). In the context of sports, sports event involvement can be defined as the knowledge and awareness of the residents of the place where the sports event are held, as well as the level of enthusiasm, interest and participation in the sports event (10). Applied to the field of sports, sports event involvement refers to the knowledge and awareness of residents in the place where a sports event is held, the degree of motivation, interest and participation in the sports event, as well as the degree of satisfaction, pleasure, and pursuit of one's own value that results from participation in a particular sports event (10, 11). Kim & Kaplanido view this as an assessment of the degree to which sports activities are at the extent to which it occupies a central place in a person's life, including watching and attending various sporting events; Beaton et al. argue that sports participation occurs when individuals make participation in sports a central part of their lives and provides hedonic and symbolic value. During the Asian Games, Hangzhou residents may be affected by a variety of direct or indirect impacts as a result of the hosting of the Asian Games. This study introduces the variable of involvement to capture the degree of Hangzhou residents' relevance to the Asian Games.

### 1.2 National identity

Identity is a multidimensional concept, which in the field of psychology refers to the differences, characteristics and their sense of belonging formed in the interaction of individuals and groups (12). National identity is people's knowledge and acceptance of their membership of the state, and it is a complex psychological structure system including political identity, cultural identity and national identity and other components (13), which promotes citizens' sense of belonging and loyalty to the country and makes them willing to contribute to the development and interests of the country (14). He believes that national identity refers to the citizens of a country's recognition of their homeland's historical and cultural traditions, moral values, ideals and beliefs, national sovereignty, etc., i.e., national identity (15). Liu believes that national identity is a feeling of similarity, equality, and intimacy among members of a country and people's feelings and evaluations of their own or other countries, etc., which is a kind of subjective consciousness and attitudes, and is the result of the historical development of a country and the process of socialization of individuals (16). In this paper, national identity mainly refers to citizens' self-awareness of their own national identity and their status and role in the international system, and it is the intersubjective social identity formed by the interaction between a country and other countries under the constraints of the international system (17).

### 1.3 City image

The concept of the city image was first proposed by Kevin Lynch, and he divided the term into five elements: roads, edges, areas, nodes and signs. Lynch argued that the city image is the mental imagery and subjective feelings formed by people's perception of the physical environment (18). The concept of city image is divided into two distinct categories: physical image and virtual image. The physical image represents a city's landscape in a generalized manner, whereas the virtual image encompasses the collective beliefs and impressions of the public within and outside the city, as perceived from the perspective of cognitive psychology (19). Liu et al. point out that subjective happiness is evaluated by individuals themselves and consists of three dimensions: life satisfaction, negative emotion, and positive emotion (20). Xing regarded subjective well-being as a psychological experience and an ideal state of existence, believing that happiness is a subjective reflection of real life, which is closely related to the objective conditions of people's lives, and also reflects people's needs and values, and that people's subjective well-being is precisely the individual's positive psychological experience of his or her own state of existence, which is produced by the joint action of these factors (21). This study considers the concept of city image as a psychological perception of the “virtual image” of the city, as influenced by improvements in city quality, city governance, city civilization and city image during the hosting of the Asian Games in Hangzhou. And that city image is the sum of the public's impressions, views and perceptions of a large amount of raw data on city layout, city environment, city culture, etc., processed and refined (22).

### 1.4 Subjective well-being

Subjective well-being is an important indicator of people's mental health, life happiness and social development. Studies related to subjective well-being have been conducted in the academic community since the 1940s, with most scholars focusing on how and why people experience their lives in positive ways. Among them, Andrews and Diener identified two aspects of subjective well-being: cognitive judgments of life satisfaction and emotional judgments consisting of separate positive and negative emotions, respectively (23). Ryff started from six different dimensions of the theoretical model of psychological well-being, i.e., autonomy, environmental mastery, personal growth, positive relationships with others, life goals, self-acceptance and self-reliance. Positive relationships, life goals, and self-acceptance, and explored the determinants of well-being through a survey (24).

In summary, the academic community has achieved rich academic research results on the subjective well-being, city image, national identity, such as the definition of the concept and associated factors, but more researchers discuss the positive impact of tournament hosting on national identity by way of theoretical analysis, and there is a lack of research on how tournament hosting affects national identity in terms of the internal paths and the degree of impact, and fewer researchers have tested the relationship between the four by constructing a model. Few researchers have examined the relationship between the four by constructing a model in an empirical way. In this paper, we take the Hangzhou Asian Games as an opportunity to explore how the Hangzhou Asian Games affects residents' sense of national identity from the perspective of residents' involvement in the event, and analyze the relationship between national identity and participation in the event by examining the relationship based on the theoretical model of involvement—mediators—national identity and relying on the data obtained from the questionnaire survey. Based on the theoretical model of involvement—mediating factors—national identity, the data from the questionnaire survey will be used to test the correlation between the two, and analyze the key factors and logic of residents' national identity, so as to provide new perspectives for the discussion of the impact of sports events on residents' national identity, and to provide practical management suggestions for the managers of the events.

## 2 Research hypothesis

### 2.1 The impact of sports event involvement on national identity

In event host territories, residents are the most direct stakeholders of the event. Each territory's residents have varying degrees of involvement in the event. Research has found that residents who are highly involved in sport may contribute to positive associations between sporting events and the behavioral intentions of community members (25). Iwasaki posits that individuals may derive a sense of self-identity from their involvement in leisure activities (26). Kim pointed out that people who participate more actively have a more positive view of the host country. This indicates that participation in activities plays an important role in shaping the image of the host country. In addition, the scholar suggests incorporating participation in sports events into the research framework to understand how events affect the impression of the host country (27). The development of sports events attracts an increasing number of people to participate in them. Those who are highly involved can gain a greater sense of national cohesion and pride from them. Furthermore, the expanding influence of the events also improves the recognition of China's comprehensive national strength by various circles at home and abroad. Therefore, this paper introduces the variable of involvement to reflect the degree of Hangzhou residents and the Asian Games, with the aim of measuring the effect of the Asian Games on residents' participation.

In conclusion, the following hypotheses are proposed:

H1: The degree of involvement in the Hangzhou Asian Games has a significant positive effect on national identity.

### 2.2 The mediating role of subjective well-being

As societal norms evolve, the value placed on happiness is on the rise. Sport, as a pro-people activity, has the potential to enhance subjective well-being. Sato examined the relationship between running sport involvement and life satisfaction. The findings suggest that psychological involvement in running sport significantly affects life satisfaction. This indicates that sport can improve the quality of life and subjective well-being through leisure involvement (28). Paul Dolan et al. found that the London Olympics increased the life satisfaction and well-being of Londoners in the short term, with a particular impact on the subjective well-being of the host country's citizens (29). Huang Ying et al. demonstrated through questionnaires and structural equation modelling that residents' participation in major sporting events has a direct positive effect on subjective well-being. Furthermore, participation affects subjective well-being through three mediators, including the quality of life in the community, and five indirect paths of action (30). Zhang Hui and Luo Jianying constructed a theoretical model of the relationship between marathon race culture and the happiness index of city residents. Their findings indicated that marathon race culture acts on the physical and mental health of residents through the material culture phenomenon that it forms, thus enhancing happiness (31). Zhang Yong et al. demonstrated that sports interactions (participation in social sports activities, sports tourism) can foster a favorable cultural atmosphere for sports events, laying the foundation for the establishment of a positive sense of subjective well-being. Furthermore, sports participation can directly enhance subjective well-being, with greater involvement leading to greater subjective well-being (32).

A significant positive effect of national identity on subjective well-being has been demonstrated by the majority of scholars (33, 34). Furthermore, some scholars have demonstrated that national identity and subjective well-being can be significantly positively predicted by each other (35). Su-Lan Pan et al. investigated the relationship between leisure participation and well-being and the mediating role of occupation-related characteristics, organizational commitment on well-being among 406 fans. A study of a BE baseball team in Taiwan revealed that tournament participants' well-being led to a positive effect on their loyalty to the participating team (36). Katharine et al. demonstrated that social identities can satisfy or impede a range of psychological needs, including belonging and well-being (37). In other words, the acquisition of national identity and identification increases a person's well-being. Wang et al. Wisdom conducted a questionnaire survey on the residents of the area hosting the 2008 Beijing Olympic Games by developing happiness index indicators. The results demonstrated that the hosting of the Olympic Games effectively improved the happiness index of Beijing citizens and stimulated people's patriotism and national pride (38).

Sports events enhance the sense of acquisition and well-being, and also enable the people to intuitively feel and construct a sense of social belonging and identity, therefore, the subjective sense of well-being and identity of the residents may be improved to a certain extent under the influence of sports events.

In conclusion, the following hypotheses are proposed:

H2: Hangzhou Asian Games involvement has a significant positive effect on subjective well-being.
H3: Subjective well-being has a significant positive effect on national identity.

### 2.3 The mediating role of subjective well-being

Sporting events represent a significant opportunity to enhance the city's image and shape its brand (39). In recent years, the success of China's sports events has made a significant contribution to the creation of local city image. This is exemplified by the successful hosting of the Beijing Winter Olympics, which has led to Beijing being dubbed the “city of the two Olympics” and has accumulated valuable experience for China in the creation of a world-renowned sports image. China has gained considerable practical experience in creating a world-renowned sports image (40). Two major international sports events, the F1 Chinese Grand Prix and the Shanghai ATP1000 Tennis Masters, have had a significant positive impact on Shanghai's city image (41). Scholars such as Wang Min and others employed the Kelly square method to investigate the audience's perceptual experience of regional elements. Their findings indicated that sports events have become an integral component of regional branding, and that they play a pivotal role in disseminating regional branding and enhancing regional image (42).

The city's natural and humanistic attractions, as well as its order and law, serve to concentrate the city residents' vernacular cognition and pride. These symbolic symbols of the city's image become the city's imagination, and then realise the emotional identity (43). Zhai Lianfen posits that the city image constitutes the focal point of the city's soft environment. He further asserts that a positive city image will significantly enhance the city's visibility and reputation, which in turn will stimulate the inflow of foreign population and stimulate the citizens' sense of community (44). Shi Fei maintains that a positive city image can increase residents' pride and cohesion, and that it is one of the most important guarantees for the harmonious and sustainable development of the city (45). Zhang Hui and colleagues demonstrated that participation in sporting events can positively influence individuals' perceptions of city identity (46). Sun Wei's investigation of the Tianjin National Games revealed that the event's conceptual, participation and health images enhanced the city's image and strengthened the public's sense of identity, belonging and pride in Tianjin (47).

A significant correlation and coupling is evident between the sports event system and the city development system. This indicates that sports events and the components of the city can interact and influence each other (48). Furthermore, successful sports events have a great impact on the improvement of city infrastructure, living environment and even the quality of life of the residents. Chen Jiaqi and colleagues posit that quality of life, life satisfaction, and happiness are mutually reinforcing and examine the impact of the Nanjing Youth Olympic Games on the city of Nanjing. Their findings indicate that the hosting of large-scale sporting events can promote city development, enhance the city's image, improve the living conditions of residents, and significantly contribute to the enhancement of residents' sense of well-being (49, 50). Fan Jing's findings indicate that the Changchun International Vasaloppet Ski Festival brought more advantages than disadvantages to the city of Changchun. Furthermore, the improvement of the city's image positively affected the subjective well-being of the residents of the territory (51).

It has become a consensus among scholars that sports events can have a wide range of impacts. These impacts can be both explicit and implicit. With regard to the latter, it can be argued that sports events have an impact on the image of the host city. This, in turn, can improve the residents' sense of well-being and cohesion. Consequently, the city's image and subjective sense of well-being may have a positive impact on the residents' sense of national identity.

In conclusion, the following hypotheses are proposed:

H4: Residents of Hangzhou who are involved in the Asian Games have a significant positive effect on the city's image.
H5: The city image has a significant positive effect on national identity.
H6: The city image has a significant positive effect on subjective well-being.

**Figure 1:**
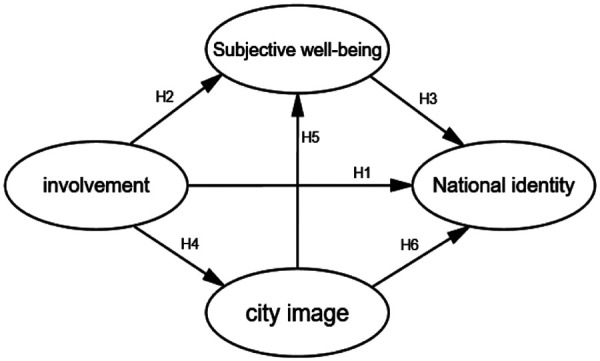
Theoretical hypothesis model.

Through the analysis and research hypotheses in the literature review section, the model of the four main variables of involvement, subjective well-being, city image, and national identity was finally formed (Figure 1). This study explores the impact of major sports events on residents' national identity from the perspective of residents' involvement in the Asian Games, and uses subjective well-being and city image as mediating variables to validate the interactions between the variables and their extent.

Summarizing the previous section, this paper proposes the following hypotheses:

H1: Hangzhou residents' Asian Games involvement has a significant positive effect on national identity.
H2: Hangzhou residents' involvement in the Asian Games has a significant positive effect on subjective well-being.
H3: Subjective well-being has a significant positive effect on national identity.
H4: Hangzhou residents' Asian Games involvement has a significant positive effect on city image.
H5: city image has a significant positive effect on subjective well-being.
H6: city image has a significant positive effect on national identity.

## 3 Method

### 3.1 Pre-survey

Recruitment began on May 15, 2023 for this study. First, the first batch of questionnaires were distributed to 420 residents of Hangzhou from May 30 to June 30, 2023, and the data collected were used for the reliability test of the questionnaires. Second, a revised pre-survey questionnaire was used to survey 1,200 randomly selected Hangzhou residents from October 10, 2023 to November 30, 2023. All citizens were fully informed about possible issues related to the research process. The study procedures were approved by the Research Ethics Committee of Hangzhou Normal University (No. 2023-0501, May 12, 2023). All the participants gave written informed consent. After excluding invalid questionnaires, 394 valid questionnaires were obtained, representing a 93.8% validity rate. Among the respondents, 183 were male (46%) and 211 were female (53%). The proportion of respondents in each age group was as follows: under 18 (17%), 18–30 (28%), 30–50 (28.9%), and over 50 (26.2%). The pre-survey data should be analyzed and the formal survey scale determined by revising the items and other methods.

### 3.2 Measurement tools

The initial scales for engagement, city image, subjective well-being, and national identity were constructed using existing mature scales. All scales were in the form of Likert 5-level scales, with 1 indicating “strongly agree” and 5 indicating “strongly disagree”.

#### 3.2.1 Involvement scale

The involvement scale was primarily derived from the study of Fengjun Zhang et al. (10), in which six items were selected. These included “It is important for me to pay attention to the event”, “Paying attention to the event is one of my main ways of relaxation”, “I pay a lot of attention to information about the event”, “Spending time to learn about the event is worthwhile for me”, “My paying attention to the event reflects my interest”, and so on. Exploratory factor analysis and principal component analysis were used to perform orthogonal rotation factor analysis on the pre-survey data, and it was found that the factor loading coefficient of “paying attention to this competition reflects my value orientation” was less than 0.6, so it was deleted. The revised inclusion measurement table consists of 5 questions, with a Cronbach coefficient of 0.912 and a KMO of 0.892.

#### 3.2.2 National identity scale

The National Identity Scale is primarily derived from Peter Gries' study (52), which is divided into 4 dimensions: patriotism, blind patriotism, nationalism, and internationalism. The scale comprises a total of 10 items, including the following statement: “I am glad to be Chinese”, “Being Chinese is an important reflection of who I am”, “China is the best country in the world”, and “Given China's long history and splendid civilization, it is natural that China should have more influence in East Asia”, and so on. The exploratory factor analysis and principal component analysis conducted on the pre-survey data using orthogonal rotated factor analysis revealed that the factor loading coefficients of “The problems faced by poor countries should be solved by themselves and have nothing to do with us” and “I regret being Chinese” were less than 0.6, indicating that these items should be deleted. Similarly, “I regret that I am Chinese” was found to be less than 0.6, and thus also deleted. The revised National Identity Scale comprises 8 items, with a Cronbach coefficient of 0.846 and a KMO of 0.796.

#### 3.2.3 Subjective well-being scale

The Subjective Well-Being Scale is a self-report instrument that measures an individual's subjective well-being. It is a 20-item scale, developed by Xing (53), with a total of 20 items, including “Society provides people with more and more ways out”, “I have learned many philosophies from life, which makes me more determined and capable”, “When encountering unpleasant things, I cannot keep my spirits up for a long time”, and so on. Exploratory factor analysis and principal component analysis were used to perform orthogonal rotation factor analysis on the pre-survey data, and the non-compliant items of factor loading coefficient, such as “I am quite satisfied with my personality”, “I sometimes find it difficult to communicate with my family (including parents, children, loved ones, etc.)”, and “I feel particularly happy when I am with my family”, were removed. The revised subjective well-being scale consists of 17 questions, with a Cronbach coefficient of 0.814 and a KMO of 0.866.

#### 3.2.4 City image scale

The city image scale is primarily derived from Huang Haiyan's Semantic Difference Scale of city image (54), which is divided into 2 dimensions: cognitive image and affective image. This study focuses on the cognitive aspect of city image, and the cognitive image dimension was selected to obtain the data necessary to answer the research question in an efficient manner, while also avoiding overlap with the Subjective Well-being Scale. The cognitive image dimension comprises 5 items, including “traditional or modern”, “low visibility or high visibility”, and “regional or international”, “atmosphere not unique or atmosphere very unique”, and so on. The final scale exhibited a Cronbach coefficient of 0.877 and a KMO of 0.873.

### 3.3 Data survey

The revised pre-survey scale was used as the official questionnaire, and a random sampling method was adopted to select 1,200 Hangzhou residents as the respondents for the questionnaire survey within 2 months after the Hangzhou Asian Games were held (October to December 2023) in the city area of Hangzhou City, where the survey areas included Xihu District, Yuhang District, Binjiang District, Gongshu District, Qiantang District, etc., Gongshu District, Qiantang District, etc., in which the survey respondents include teachers, students and residents of Hangzhou Normal University, Zhejiang University, Zhejiang University of Metrology, Zhejiang University of Traditional Chinese Medicine and other universities and their affiliated primary and secondary schools, communities. The Tencent questionnaire was filled out online for college students, office workers and other young and middle-aged people; the questionnaire was filled out by primary and secondary school students through visits to campuses; and the questionnaire was read aloud to middle-aged and elderly people with a low level of education, so that they could fully understand the meaning of the questionnaire and then fill it out truthfully. The research ethics procedure was consistent with the pre-survey, and all the participants gave written informed consent. The ethical procedures of the study were consistent with the pre-investigation and all participants signed an informed consent form. Finally, excluding the invalid questionnaires with problems such as not filling in the questionnaires carefully and logical errors, 1,096 valid questionnaires were collected and collated, and the validity of the questionnaires was 91.3%. Among the valid questionnaires, 528 were male, accounting for 48.2%, and 568 were female, accounting for 51.8%. The proportion of respondents in each age group was as follows: under 18 (24.8%), 18–30 (41.7%), 30–50 (20.8%), and over 50 (12.7%) (Table 1).

**Table 1:** Description of sample feature distribution.

Variable	Option	Frequency	Percentage
Gender	Male	528	48.20
	Female	568	51.80
Age	<18	272	24.80
	18–24	332	30.30
	25–30	125	11.40
	31–40	104	9.50
	41–50	124	11.30
	51–60	111	10.10
	>60	28	2.60
Educational background	Junior high school and below	254	23.20
	High school	103	9.40
	Undergraduate	490	44.70
	Postgraduate and above	249	22.70
Time in Hangzhou	Less than 1 year	96	8.80
	1–3 year	149	13.60
	3–10 year	268	24.50
	Over 10 years	583	53.20

### 3.4 Reliability testing

The internal consistency of the dimensions was analyzed using the Cronbach coefficient reliability test method in SPSS 27.0, with the reliability coefficient values observed to be between 0.853 and 0.928 (Table 2). These values exceed the criterion of 0.8, indicating that the measurement questionnaire has a high level of reliability.

**Table 2:** Reliability and validity analysis.

Index	Cronbach's α	KMO	sig	CR	AVE
Involvement	0.928	0.892	0.00	0.929	0.723
National identity	0.855	0.876	0.00	0.872	0.464
SWB	0.899	0.911	0.00	0.900	0.353
City image	0.853	0.851	0.00	0.859	0.551

SPSS 27.0 was employed to execute the KMO and Bartlett's spherical test on the scale. The KMO value was found to be greater than 0.7, indicating that the scale exhibited a high degree of reliability. Furthermore, the closer the value approached 1, the higher the reliability. Additionally, the *P* value of the Bartlett's spherical test was found to be less than 0.01, signifying a significant correlation between the variables. The results of the analysis indicated that the KMO values of the questionnaire ranged from 0.851 to 0.911, all of which were greater than 0.8. Furthermore, the *p*-values were all 0.00, less than 0.01, suggesting that the data from the questionnaire were well suited for factor analysis (Table 2).

The average variance extraction (AVE) and construct reliability (CR) of each measurement item on the corresponding dimension were calculated using the confirmatory factor analysis (CFA) model established by AMOS 24.0. This was done through validated factor analysis. According to the established criteria, an AVE value of 0.36–0.5 is deemed acceptable, a value exceeding 0.5 is considered excellent, and a CR value of 0.7 is regarded as excellent. Among these, the AVE value of the subjective well-being scale is marginally below the acceptable range. This may be attributed to the scale's excessive number of items, but the high CR value (0.9) and good discriminant validity (Table 3) indicate that the scale's validity remains acceptable (55, 56). The AVE values of the remaining scales exceeded 0.46, while the CR values exceeded 0.8, indicating satisfactory convergent validity and combinatorial validity among the dimensions of the scales (Table 2). In the differential validity test, the standardized correlation coefficients between the dimensions were less than the square root of the AVE value corresponding to the dimension, indicating satisfactory differential validity between the dimensions (Table 3). In conclusion, the questionnaire and its dimensions exhibited satisfactory reliability and validity in this study.

**Table 3:** Differential validity analysis.

Index	Involvement	National identity	SWB	City image
Involvement	0.850			
National identity	0.302	0.742		
SWB	0.325	0.329	0.594	
City image	0.442	0.350	0.475	0.681

SWB, subjective well-being.

### 3.5 Statistics

This study employs mathematical statistics and model validation through SPSS27.0 and AMOS24.0 software, as well as structural equation modelling. Descriptive statistics and correlation analyses of the scales were conducted initially, followed by validation factor analysis and model fit testing. This involved the use of packing and the establishment of correlation paths to improve model fit, as well as testing the path coefficients of each hypothesis. Finally, bootstrap was employed to test the mediating effect and analyze the research hypotheses.

## 4 Results

### 4.1 Descriptive statistics and correlation analysis

The results of the descriptive statistics analysis and correlation analysis (Table 4) indicate that the mean score of each variable is between 3.6 and 4.5, with a scoring method of 1–5. This suggests that the level of data in this study is above the medium range. Correlation analysis allows for the identification of the direction and degree of correlation between each variable. In this analysis, a correlation is observed between each variable, with the correlation coefficient r between each variable exceeding 0, indicating that each variable in this analysis is a significant positive correlation.

**Table 4:** Descriptive statistics and correlation analysis for each dimension.

	M(SD)	Involvement	National identity	SWB	City image
Involvement	3.61 (1.01)	1			
National identity	4.52 (0.53)	0.402**	1		
SWB	4.01 (0.62)	0.340**	0.434**	1	
City image	4.36 (0.61)	0.277**	0.315**	0.324**	1

Note: **At the 0.01 level (double tailed), the correlation is significant. SWB, subjective well-being.

### 4.2 Model fit test and correction

In this study, the model fitness is evaluated using the following statistical indices: CMIN, RMSEA, GFI and CFI. In order to make the model concise and reduce errors, this research employed the *a priori* questionnaire method (57) based on the strategy of unique information packaging. The subjective well-being scale, comprising 17 items, was then packaged into three indicators (58) according to its component structure, namely, health experience, contentment experience, and developmental experience. These indicators were subsequently used for structural equation modelling. The results indicate that the chi-square degrees of freedom ratio of the overall model is slightly higher than 5, with the exception of the remaining indicators, which are within the acceptable range (Table 5, uncorrected results). In order to enhance the model's fit, model correction will be conducted in this study.

**Table 5:** Model fitness test.

Index	Uncorrected/corrected result	Reference standards (59)
CMIN	1,010.670/628.158	CMIN/DF ratio 1–3 is excellent, 3–5 is good
DF	183/177	
CMIN/DF	5.523/3.549	
RMSEA	0.064/0.048	<0.05 is excellent, <0.1 is good
GFI	0.917/0.947	>0.9 is excellent, >0.8 is good
CFI	0.936/0.965	>0.9 is excellent, >0.8 is good

**Figure 2:**
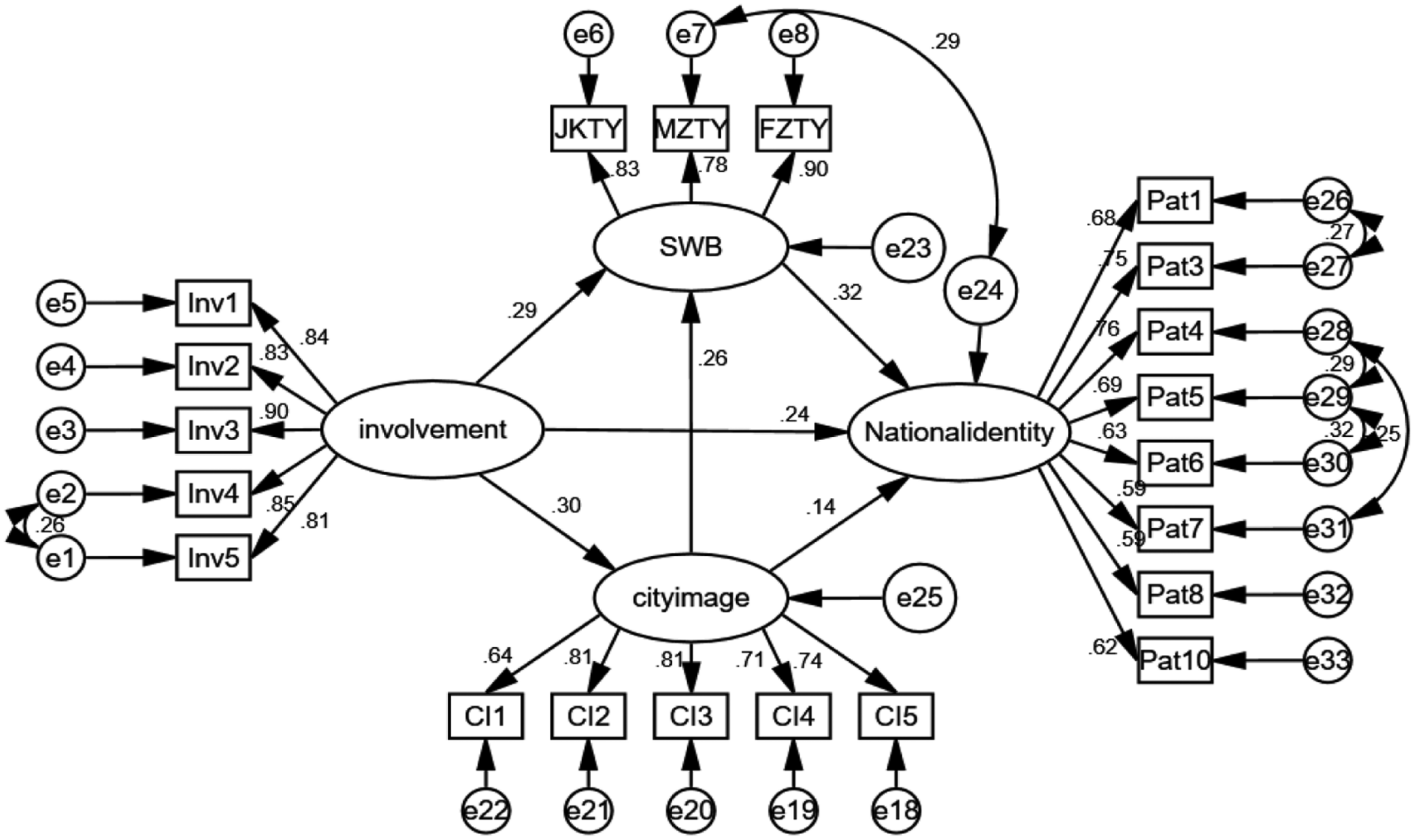
Test results of path relationship hypothesis in structural equation model.

The AMOS 24.0 software was employed for model fit and path analysis, with the following paths being proposed according to the correction index: e1–e2, e7–e24, e26–e27, e28–e29, e28–e31, and e29–e30 (Figure 2). The correction results show that RMSEA = 0.048, GFI = 0.947, and CFI = 0.965 (Table 5), all indicators have reached an excellent level. The chi-square degrees of freedom ratio is 3.549, which may be attributed to the survey of Hangzhou residents with a large sample capacity influencing the CMIN value and its test results. However, given the model's excellent absolute fit index and the susceptibility of the chi-square degrees of freedom ratio to the characteristics of the sample capacity, the ratio is slightly higher than 3, which is acceptable (59). Overall, the model of this study has a good fitness for purpose.

### 4.3 The direct role of sports event involvement, city image, and subjective well-being on national identity

The results of the modified SEM standardized path relationship and hypothesis test (Table 6) indicate that the standardized path coefficient of involvement degree acting on national identity is 0.237 (*P* < 0.001); the standardized path coefficient of involvement degree acting on subjective well-being is 0.287 (*P* < 0.001); and the standardized path coefficient of involvement degree acting on city image is 0.3 (*P* < 0.001). These findings indicate that Hangzhou residents' Asian Games involvement has a significant positive effect on national identity, subjective well-being and city image. Consequently, hypotheses H1, H2 and H4 are valid. The standardized path coefficient of subjective well-being acting on national identity is 0.321 (*P* = 0.001); the standardized path coefficient of city image acting on national identity is 0.141(*P* < 0.001). This indicates that both subjective well-being and city image have a significant positive effect on national identity, thereby establishing hypotheses H3 and H6. Furthermore, the standardized path coefficient of city image on subjective well-being is 0.264 (*P* < 0.001), indicating that city image has a significant positive effect on subjective well-being. Hypothesis H5 is thus confirmed. In conclusion, the modified structural equation model has validated all the hypotheses of this study.

**Table 6:** SEM path relationship test results of influencing factors.

Path relationship	Estimate	S.E.	C.R.	*P*
H1: Involvement→National identity	0.237	0.012	7.097	***
H2: Involvement→Subjective well-being	0.287	0.022	8.602	***
H3: Subjective well-being→National identity	0.321	0.02	8.633	***
H4: Involvement→City image	0.3	0.02	8.816	***
H5: City image→Subjective well-being	0.264	0.039	7.53	***
H6: City image→National identity	0.141	0.019	4.236	***

Note: *** Denotes *p* < 0.001.

### 4.4 The mediating role of city image and subjective well-being

A bootstrap was employed to assess the mediating role of subjective well-being and city image. A total of 2,000 sample tests were conducted, resulting in the calculation of direct, indirect, total indirect, chained mediating and total effects. The findings are presented in Table 7. The bias-corrected 95% CI confidence interval for the direct effect (involvement → national identity) was 0.052–0.115, excluding 0, indicating that the direct effect of involvement on national identity was significant and the strength of the effect was 0.082. The bias-corrected 95% CI for the indirect effects (involvement → subjective well-being → national identity, involvement → city image → national identity) was 0.082, and the confidence interval for the indirect effect was 0.052–0.115. The 95% confidence intervals for the indirect effects were 0.018–0.049 and 0.006–0.025, respectively, with none of the values being equal to zero. This suggests that subjective well-being and city image play a mediating role in the model, and that their total indirect effect is 0.046, which is significant. The bias-corrected 95% CI confidence interval for the chain mediation effect (involvement → city image → subjective well-being → national identity) was 0.005–0.013, not including 0, indicating the existence of the chain mediation effect in the model. The deviation-corrected 95% CI confidence interval for the total effect is 0.099–0.179, not including 0, indicating that the total effect is significant and the strength of the effect is 0.137. The results indicate that both the direct effect and the mediating effect are present in the model. The direct effect (0.082) is greater than the indirect effect (0.046), suggesting that the mediating effect of subjective well-being and city image is a partial mediating effect. Furthermore, the involvement of Hangzhou residents in the Asian Games has a significant direct effect on national identity.

**Table 7:** Structural model mediation effect test.

Path effect	Strength	Bias-corrected 95% CI confidence interval		*p*
		Lower	Upper	
Direct effects	0.082	0.052	0.115	0.001
Indirect effects	0.032	0.018	0.049	0.001
Indirect effects 2	0.015	0.006	0.025	0.002
Total indirect effect	0.046	0.032	0.063	0.001
Chain mediation effect	0.009	0.005	0.013	0.001
Aggregate effect	0.137	0.099	0.179	0.001

## 5 Discussion

This study examines the impact of Hangzhou residents' involvement in the Asian Games on their national identity. A structural equation model is constructed to analyze the interrelationship between subjective well-being and city image as mediating variables. The model fit of this study was relatively good (CMIN/DF = 3.549) and all indicators were excellent (RMSEA = 0.048, GFI = 0.947, CFI = 0.965) and all the hypotheses of this study were confirmed by structural equation modeling. Specifically, involvement has a significant positive effect on national identity, subjective well-being and city image. Both subjective well-being and city image have a significant positive effect on national identity, and city image has a significant positive effect on subjective well-being. Furthermore, the bootstrap test for mediating effects indicates that the direct effect of involvement on national identity is significant, that subjective well-being and city image play a partial mediating role in the model, and that there is a chain mediating effect in the model.

### 5.1 The impact of Asian games involvement on national identity

The analysis results show that the involvement of Hangzhou residents in the Asian Games has a significant positive effect on national identity (standardized path coefficient of 0.237, *P* < 0.001), i.e., the more Hangzhou residents are involved in the Asian Games events, the stronger the sense of national identity will emerge from them, which is consistent with the conclusions of previous scholars’ studies (13, 27) and verifies hypothesis H1. The Hangzhou Asian Games involve a large number of people, with all the members of the Olympic Games Committee and 12,000 athletes registered to participate, and more than 130 countries and regions with more than 100 million people participating in the Games. Council and 12,000 athletes registered to participate in the Games, more than 130 countries and regions more than 100 million people involved in watching the Games, ticket sales exceeded 600 million yuan, there are also 37,600 volunteers on and off the field to provide services, people through a variety of forms of involvement in the Games (60). As the largest Asian Games ever held with unprecedented standards, the Hangzhou Asian Games featured 40 main events, 61 sub-events and 481 sub-activities (61), demonstrating China's national self-confidence to the world in every aspect: The Hangzhou Asian Games demonstrated China's national self-confidence to the world in every aspect: pioneering digital technology to promote China's traditional cultural heritage and future technology to the world, stimulating the audience's empathy for the motherland through the course ceremonies, infectious music and participatory interactive ways at the venue; and stimulating the audience's empathy for the motherland through the dissemination of vivid and three-dimensional media. The event media enhanced the public's understanding and recognition of China's culture through the dissemination of vivid and three-dimensional images of China, which further enhanced the country's image in the minds of those involved in the Asian Games. Furthermore, Hangzhou residents participated in the Asian Games events, experiencing the grandeur of the Hangzhou Asian Games and the charm of the Hangzhou Asian Games in terms of mathematics and intelligence. This enhanced the residents' sense of honour in participating in the Asian Games, promoted their perception of the national image, and thus enhanced their sense of belonging to the country and national identity.

### 5.2 The mediating role of subjective well-being

The results of the analysis show that Hangzhou residents' involvement in the Asian Games has a significant positive effect on subjective well-being (standardized path coefficient of 0.287, *P* < 0.001), and subjective well-being has a similarly significant positive effect on national identity (standardized path coefficient of 0.321, *P* < 0.001), suggesting that Hangzhou residents' involvement in the Asian Games can not only directly affect national identity, but also indirectly affect national identity by influencing subjective well-being, in which subjective well-being plays a partial mediating role. This finding is consistent with previous research that event participants can significantly increase their subjective well-being by experiencing the event culture (31), and that there is a strong relationship between the level of involvement in major sporting events and the well-being generated by positive emotions as well as the sense of national identity (62). Residents' well-being is largely derived from quality of life, social support and sense of belonging. The Hangzhou Asian Games allowed people to experience changes in the quality of life around them. During the preparation period, Hangzhou stir up a national sports boom, 56 competition venues are all open to the public, “Asian Games venues online venues” has been 384 venues, of which 45 Asian Games venues, the total number of people in fitness 20 million people, the total number of orders 1.15 million (5). In the post-Asian Games period, Hangzhou is committed to transforming the legacy of the Asian Games into sustainable value, enabling digital empowerment of sports, and citizens show the confidence of “everyone is a host”, and the sense of well-being brought by the Asian Games is emerging. This study suggests that the organizers of the event can fully implement the “fitness for all” policy in the context of building a sports power, and increase residents' involvement in the event by opening sports venues, organizing small-scale sports activities, recruiting volunteers, and enriching the channels for watching the event, so that residents can fully feel the atmosphere of the event and the changes in the city, thereby enhancing their subjective sense of well-being and national identity. This will enhance residents' subjective well-being and national identity.

### 5.3 The mediating role of city image

The analysis results show that Hangzhou residents' involvement in the Asian Games has a significant positive effect on city image (standardized path coefficient of 0.3, *P* < 0.001), and city image has a similarly significant positive effect on national identity (standardized path coefficient of 0.141, *P* < 0.001), and in addition, involvement can also affect national identity through the chain mediating effect of city image and subjective well-being (strength of effect 0.009, *P* = 0.001), and this result proves that involvement can affect city image while also indicating that city image has a significant effect on subjective well-being (strength of effect 0.264, *P* = 0.001). 001), and this result proves that involvement can affect city image, while also indicating that city image has a significant effect on subjective well-being (strength of effect 0.264, *P* = 0.001). Identity (strength of effect 0.009, *P* = 0.001), this result proves that the degree of involvement can affect city image at the same time, it also indicates that city image can have an effect on both subjective well-being (standardized path coefficient of 0.264, *P* < 0.001) and national identity. Following the concept of sustainable development, Hangzhou adopted “green power” and water recycling system, built 31 training venues, 56 competition venues and 5 Asian Games villages, and constructed 1,588 public charging stations and more than 21,000 public charging piles. More than 21,000 public charging stations were built, fully demonstrating the image of the Asian Games City of green, low-carbon, digital intelligence and humanistic Jiangnan (63). In addition, Hangzhou held more than 140 non-legacy display activities during the Asian Games, and successively launched 10 Asian Games theme tourism lines and more than 70 Asian Games tourism concessions, so that more people can feel that Hangzhou is rich in history and culture and full of creativity (64, 65). It was learned from the visited residents involved in the Hangzhou Asian Games that the improvement of Hangzhou's city image has made them feel a strong sense of belonging and happiness, and the unprecedented expansion of Hangzhou's city brand influence has made the concept of oriental wisdom and Chinese style accepted by the world, which has an important impact on strengthening Hangzhou residents' identity of the city and the country. It can be seen that if the event organizer can solve the problems of optimizing facilities and road construction before the event so as to minimize the inconvenience to residents' lives; and do a good job of cultural output and provide quality services during the event so that those involved in the event can feel the charm of the city they live in, it will have a positive effect on the enhancement of the city's image.

### 5.4 Research limitations and future prospects

This paper explored the relationship between Hangzhou residents' Asian Games involvement, city image, subjective well-being and national identity through structural equation modeling, but there are still some limitations. First, this study mainly explores the short-term positive effects of hosting large-scale sports events on residents' national identity while ignoring the long-term effects and negative impacts. Secondly, this study takes Hangzhou residents as a whole as the target population and does not compare the differences in the impacts on different types of Hangzhou residents after they have been involved in the Asian Games. Third, this study mainly investigated two mediating variables, subjective well-being and city image, without further exploring the possibility of the existence of other moderating variables under this theoretical framework. In future research, it is possible to track and investigate the bidirectional impacts of various types of large-scale sports events on residents' national identity at different time periods, to consider the research hypothesis of counteracting effects, to analyze the impacts of different aspects of social impacts produced by large-scale sports events on residents' national identity, and to improve the model structure in order to analyze in a deeper way the internal mechanism of residents of large-scale sports events' territories constructing their national identities by getting involved in the events.

## 6 Conclusions

This study assesses involvement, subjective well-being, city image and national identity. The study uses the Hangzhou Asian Games as a case study to explore the extent and path of the impact of hosting large-scale sports events on national identity from the perspective of local residents. The findings of the study provide a resident's perspective for assessing the social impact of large-scale sports events, and also provide elements that can be used as a reference by governments and organizations in assessing large-scale sports events. This study examines the immediate positive effects of hosting events on residents' national identity, but it does not consider the long-term or negative impacts. In the future, we will conduct further research to investigate the bi-directional impacts of various types of large-scale sports events on residents' national identity in different time periods. This will enable us to enrich and improve the study.

## Data Availability

The raw data supporting the conclusions of this article will be made available by the authors, without undue reservation.
